# American Medical Association

**Published:** 1878-06

**Authors:** Wm. B. Atkinson

**Affiliations:** Permanent Secretary


### EDITORIAL.

### American Medical Association.

It now seems certain that the approaching meeting of the American Medical Association will be of more than usual interest; that the "Sections" will be better organized, and prove more instructive and valuable on that account. As Buffalo belongs to no section or party, and is but the centre of medical civilization this year, in the same sense that Boston is the "hub of the universe," it is to be hoped and expected that fewer disturbing influences will appear than has usually been observed. We as yet know of nothing to mar or disturb the true objects of the Medical Association, and in the brief space left us for editorial comment would most earnestly urge upon our readers the duty and importance of attendance upon the session—duty to the profession, and value to themselves. Hoping to see and make personal acquaintance with many formerly known only by reputation, we hereby extend a most cordial invitation to attend the approaching meeting of the American Medical Association, believing ample return for the expenditure will be received.

We give below a Directory of the Places of Meeting of the various Sections:

General Session, St. James Hall, corner of Eagle and Washington streets.

Section I.—Practical Medicine, Materia Medica and Physiology, No. 354 Washington street, 2d story.

Section II.—Obstetrics and Diseases of Women and Children, No. 354 Washington street, 3d story.

Section III.—Surgery and Anatomy, St. James Hall.

Section IV.—Medical Jurisprudence, Chemistry and Psychology, Firemen's Association Rooms, Y. M. A. Building, entrance through St. James Hall and on Main street.

Section V.—State Medicine and Public Hygiene, Baptist Church Chapel, Washington street, near Swan.

Judicial Council Room.—Y. M. A. Building, entrance from St. James Hall.

Registration Room.—No. 354 Washington street, 2d story.

Registration open from 2 to 6 P. M. on Monday, June 3d; from 8 to 11 A. M. on Tuesday; and from 8¼ to 10 A. M. on each day thereafter.

*Programme of the First General Session, Tuesday, June 4th.*—11 A. M., Introduction of the President, T. G. RICHARDSON, M. D., of New Orleans., La. Prayer, by the Rev. L. Van Bokkelen, D. D. Address of Welcome, by Thos. F. Rochester, M. D. Report of the Committee of Arrangements on the credentials of members. Reception of members by invitation, and reading of notes from absentees. Election of permanent members on the recommendation of the Committee of Arrangements. 12 M.—Hearing of the Annual Address of the President, Dr. T. G. Richardson. Call for reports of special committees and volunteer papers, and their reference to the appropriate Sections. Report on the action of the American delegation before the International Medical Congress of Geneva, on the question of International Medicine and Pharmaceutic Uniformity, by E. C. Seguin, M. D., of New York. 1 P. M.—Adjournment. At 3 P. M. each day, the Sections meet in the respective Halls assigned to their use. The whole programme is too long for publication, but can be had upon application.

FARES. From Philadelphia to Buffalo and return, one fare and a-half. From Baltimore to Philadelphia and return, $4. The Canada Southern and Wabash Railroad will give excursion tickets to delegates to the American Medical Association, and their families, for half the usual fare. They can procure tickets for the round trip by presenting their certificates of appointment, for *one* full fare.

This arrangement reduces the fare one-half from St. Louis, Toledo, and Detroit to Buffalo, through connecting lines reaching the whole Southwest.

WM. B. ATKINSON, *Permanent Secretary.*

